# Grow With the Challenge – Microbial Effects on Epithelial Proliferation, Carcinogenesis, and Cancer Therapy

**DOI:** 10.3389/fmicb.2018.02020

**Published:** 2018-09-20

**Authors:** Jakob von Frieling, Christine Fink, Jacob Hamm, Kenneth Klischies, Michael Forster, Thomas C. G. Bosch, Thomas Roeder, Philip Rosenstiel, Felix Sommer

**Affiliations:** ^1^Zoological Institute, Christian-Albrechts-Universität zu Kiel, Kiel, Germany; ^2^Institute of Clinical Molecular Biology, Christian-Albrechts-Universität zu Kiel, Kiel, Germany

**Keywords:** microbiota, proliferation, cancer, chemotherapy, xenobiotics

## Abstract

The eukaryotic host is in close contact to myriads of resident and transient microbes, which influence the crucial physiological pathways. Emerging evidence points to their role of host–microbe interactions for controlling tissue homeostasis, cell fate decisions, and regenerative capacity in epithelial barrier organs including the skin, lung, and gut. In humans and mice, it has been shown that the malignant tumors of these organs harbor an altered microbiota. Mechanistic studies have shown that the altered metabolic properties and secreted factors contribute to epithelial carcinogenesis and tumor progression. Exciting recent work points toward a crucial influence of the associated microbial communities on the response to chemotherapy and immune-check point inhibitors during cancer treatment, which suggests that the modulation of the microbiota might be a powerful tool for personalized oncology. In this article, we provide an overview of how the bacterial signals and signatures may influence epithelial homeostasis across taxa from cnidarians to vertebrates and delineate mechanisms, which might be potential targets for therapy of human diseases by either harnessing barrier integrity (infection and inflammation) or restoring uncontrolled proliferation (cancer).

## Introduction

Tissue homeostasis requires a balance between cell proliferation and cell death and thus a tight regulation of these processes. This is particularly evident in epithelial barrier organs, where a constant renewal of the cellular lining contributes to their critical function as interfaces to the environment. Dysregulation of these processes can lead to diseases, in particular infections and cancer. Cancer research traditionally focused on cell-intrinsic mechanisms and mutations that drive carcinogenesis or render tumors resistant to chemotherapy. However, cancer does not develop in isolation but in contact with other non-malignant cells and environmental factors, which together build the tumor microenvironment. In the past 10 years it has become evident that the microbiota contributes to many aspects of the regenerative response and cell death decisions of epithelia and thereby has an impact on host health. The natural microbiota modulates the resistance against mutagen/inflammation-induced colorectal tumorigenesis, and recently, several studies have reported that the microorganisms also modulate the efficacy of cancer treatment and chemotherapy. The gut microbiota metabolizes many xenobiotics, including chemotherapeutics, making some of these functional for the host while rendering other substances inactive. Licensing of anti-tumor immune responses adds to the critical function of the resident microbes. Taken together, the endogenous microbiota not only affects cellular homeostasis and cancer susceptibility but the microorganisms also have an impact on disease prognosis and the efficacy of chemotherapy. Thus, targeting the composition and activity of the microbiota represents a promising approach to prevent carcinogenesis and to boost the success rate of chemotherapy. Here, we discuss the recent advances in our understanding of how the microbiota regulates tissue homeostasis and contributes to carcinogenesis along with its implications for treatment options and outcome.

## Gut Microbes Tune Host Tissue Homeostasis

The gut microbiota has direct effects on cellular proliferation and cell death and is therefore of central importance to maintain tissue homeostasis. In germfree (GF) mice, which are devoid of any microorganisms, the intestinal crypts, the location of stem cells, are shorter and contain fewer stem cells than the crypts of conventionally raised (CONV-R) mice, that harbor a normal microbiota (Alam et al., 1994; Pull et al., 2005; Sommer et al., 2015). In zebrafish, the resident bacteria play important roles in the maturation of the intestine, including promoting intestinal epithelial cell (IEC) proliferation and recruiting innate immune cells in the gut. Many of these effects of the microbiota are conserved across animal species (Bates et al., 2006; Cheesman et al., 2011; Hill et al., 2016). Furthermore, the microorganisms also directly boost cell proliferation as the IECs of the CONV-R mice have a higher turnover rate due to an increased cellular renewal rate, increased migration from the crypt to the tip within the epithelium, and an increased apoptosis rate as compared to the GF mice (Abrams et al., 1963; Savage et al., 1981; Crawford and Gordon, 2005). This important role of the microbiota in maintaining the proliferative epithelial homeostasis is not restricted to mammals, as even in simple organisms such as the fruit fly *Drosophila melanogaster*, the intestinal stem cell activities and cell fate decisions are regulated by the indigenous microbiota. Similar to that observed in mice, the mitotic indexes and consequently the epithelial renewal rates of the GF flies are lower compared to CONV-R flies (Buchon et al., 2009a; Broderick et al., 2014). This indicates that the members of the intestinal microbiota influence the basal intestinal stem cell activity and are also highly relevant for maintaining the intestinal homeostasis and a healthy gut morphology. Furthermore, the microbiota contributes to the epithelial homeostasis by tuning the epithelial differentiation and thus changing the relative proportions of the different intestinal cell types in flies and mice (Smith et al., 2007; Sommer and Backhed, 2013; Sommer et al., 2015). The guts of GF flies have a lower relative number of enterocytes, less progenitor cells, and more enteroendocrine cells than the guts of the CONV-R flies. Deregulated Notch signaling that is observed in the intestines of the GF flies is supposed to cause this disturbed tissue homeostasis (Ohlstein and Spradling, 2006, 2007; Broderick et al., 2014). Besides the effects of the commensal microbiota on the intestinal homeostasis, even single bacterial species or molecules have already been identified that modulate the proliferative state of the host’s intestine. In *Drosophila*, infections with the pathogenic bacteria *Erwinia carotovora*, *Serratia marcescens*, *Pseudomonas entomophila*, and *Pseudomonas aeruginosa* lead to cell death, a strong mitotic response, and an infection-induced epithelial renewal, caused by the stimulation of intestinal stem cell activities (Apidianakis et al., 2009; Chatterjee and Ip, 2009; Jiang et al., 2009; Buchon et al., 2010). Multiple signaling pathways are involved in the intestinal cellular response upon confrontation with the pathogenic bacteria, including the c-Jun-N-terminal kinase (JNK) pathway, the epidermal growth factor receptor (EGFR) pathway, the immune deficiency (Imd) pathway, and the Janus kinase/signal transducer and activator of transcription (JAK-STAT) pathway (Apidianakis et al., 2009; Buchon et al., 2009a,b, 2010; Cronin et al., 2009). In mice, Toll-like receptor (TLR) signaling in response to confrontation with bacteria is required for normal tissue homeostasis (Rakoff-Nahoum et al., 2004, 2015). Surprisingly, in insects, the intestinal stem cells are highly responsive to direct immune activation through the IMD pathway (Fink et al., 2016), which is the major NF-κB pathway operative in this system. Chronic activation of this signaling pathway in the intestinal stem cells altered the Ras, JNK, and JAK-STAT signaling and induced massive hyperplasia that finally caused intestinal tumor formation. Moreover, this stem cell specific immune activation also disturbs the Notch-signaling, leading to an increase in enteroendocrine cell numbers (Petkau et al., 2017). Interaction with bacteria, especially with those having a pathogenic potential, appears to be the driving force underlying the regulation of stem cell activity. In flies, the stressed enterocytes produce the cytokine Unpaired3 (Upd3, the fly’s IL6 orthologue), which in turn triggers the JAK/STAT signaling in intestinal stem cells and progenitor cells, thereby promoting their proliferation and differentiation required for replacement of damaged enterocytes (Vodovar et al., 2005; Buchon et al., 2009a,b; Jiang et al., 2009). Consequently, the inhibition of JAK/STAT signaling in intestinal stem cells impairs the epithelial renewal and decreases the survival upon bacterial infection (Ha et al., 2005; Buchon et al., 2009a). Thus, the renewal of epithelia is a highly critical regulatory process in the intestinal response upon contact with different types of bacteria in *Drosophila* and in mammals. Understanding both, the mechanisms leading to a changed intestinal microbiota composition or function and the outcome of this disturbed homeostasis, will help to elucidate their importance for cancer development.

## Microorganisms Modulate Cancer Susceptibility and Development

Imbalanced cellular homeostasis, either due to uncontrolled cell proliferation or suppression of cell death, can result in the development of cancer (Garrett, 2015). Inflammation plays a critical role in the initiation and progression of epithelial malignancies in the gut (Blumberg and Powrie, 2012). Microorganisms modulate inflammation and thereby could impact carcinogenesis (Hope et al., 2005; Sommer and Bäckhed, 2016). Bacterial load is increased in colonic biopsies from patients with colorectal cancer or colonic adenomas (Swidsinski et al., 1998). Furthermore, dysbiosis (an altered microbiota composition or function) is associated with several diseases. Microbial diversity is regarded as an indicator of health and is decreased in patients with autoimmune diseases, obesity, diabetes, or chronic inflammatory bowel disorders (Ley et al., 2005; Qin et al., 2010; Sommer et al., 2017). However, in colorectal carcinoma the microbial diversity is increased (Sanapareddy et al., 2012; Russo et al., 2017). The dysbiotic cancer microbiota is enriched for the Gram-negative bacteria *Parvimonas*, *Peptostreptococcus*, *Prevotella*, *Bacteroides fragilis*, and *Fusobacterium nucleatum* (Castellarin et al., 2012; Kostic et al., 2012; Yu et al., 2017). In a recent study, the fecal metagenomic signatures performed as good as the standard clinical chemistry methods in the non-invasive identification of colorectal cancer patients (Zeller et al., 2014). In combination, these metagenomics markers and the standard fecal occult blood test even showed an improved sensitivity at the same level of specificity, thus improving the accuracy of diagnosis. Emerging data suggest that certain groups of bacteria might promote whereas others might protect against colon cancer (Couturier-Maillard et al., 2013; Radulovic et al., 2018; Schmidt et al., 2018). Indeed, *F. nucleatum* seems to play a central role in the tumor microenvironment as its abundance correlates with cancer progression as well as the dysbiotic tumor microbiota composition including *Bacteroides*, *Selenomonas*, and *Prevotella* species. Furthermore, *Fusobacterium* spreads along with metastatic tumors, and treatment with the antibiotic metronidazole reduced the *Fusobacterium* load and also cancer cell proliferation and tumorigenesis (Bullman et al., 2017). Interestingly, a recent study showed that exposing laboratory mice to a natural microbiome found in the wild mice protects against mutagen- and inflammation-induced colorectal tumorigenesis (Rosshart et al., 2017). The GF mice colonized with a natural microbiota had a significantly lower number of colorectal tumors than mice colonized with a microbiota from the standard laboratory mice (Rosshart et al., 2017). The observation that a natural gut microbiome can confer protection from colitis-associated tumorigenesis is of immense relevance since colorectal cancer represents a significant disease burden in humans (Torre et al., 2015).

Mechanistically, specific bacterial products might be accountable for the effects induced by different microbiota entities on cancer development. Bacterial toxins can induce DNA damage responses and genomic instability in host cells, and virulence factors can trigger host pathways important for carcinogenesis and inflammation (Garrett, 2015). Microorganisms or their metabolites have in fact been used as a cancer therapeutic for a long time. Already in 1891, a combination of the toxins from *Streptococcus erysipelas* and *Bacillus prodigiosus* were successfully used to treat sarcoma (Coley, 1910), and even today the mycobacteria are used as a therapeutic for bladder cancer (Lamm et al., 2014). Notably, due to their divergent microbial communities, mice of the same genetically identical strain but from different facilities (Jackson Laboratory or Taconic Farms) displayed differential tumor growth, which was equalized by co-housing or fecal transfer (Sivan et al., 2015). Few other bacterial species have already been shown to functionally modulate carcinogenesis. Colonization of GF mice with *B. fragilis*, which is also carried by asymptomatic individuals, promotes carcinogenesis (Toprak et al., 2006; Wu et al., 2007). Enterotoxigenic *B. fragilis* produce a toxin, which promotes the production of reactive oxygen species by the colonic epithelium that, in turn, can damage the DNA, cause mutations, and ultimately lead to carcinogenesis (Goodwin et al., 2011; Boleij et al., 2015). An increased carcinogen production is associated with an enrichment of aerobic bacteria and can be lowered by the *Lactobacillus* species (Chung et al., 1992). *Streptococcus gallolyticus* induces cyclooxygenase 2 expression, which is associated with disease progression (Ogino et al., 2008; Abdulamir et al., 2011). *Fusobacterium nucleatum* is probably the best-studied cancer-associated microorganism. *F. nucleatum* is not only enriched in cancer, its abundance increases with the tumor stage and has been associated with a more advanced disease status in colorectal cancer patients (Castellarin et al., 2012; Kostic et al., 2012). Mechanistically, *F. nucleatum* binds to E-cadherin of host epithelial cells via its adhesion protein FadA, which stimulates Wnt/β-catenin signaling and thereby promotes epithelial transformation (Rubinstein et al., 2013). Furthermore, *F. nucleatum* also induces an infiltration of proinflammatory immune cells into the tumor tissue and thereby elicits carcinogenic immune responses (Kostic et al., 2013; Park et al., 2014; Garrett, 2015). At the same time, *F. nucleatum* also modulates the host’s natural killer (NK) cells by direct binding of its Fap2 lectin to TIGIT, an inhibitory receptor present on all human NK cells, which leads to blunting of NK cell cytotoxicity (Gur et al., 2015). Furthermore, several other opportunistic pathogens, such as *Helicobacter pylori* and *Salmonella enterica*, promote cancer development. This topic is extensively covered by excellent recent publications to which we refer to for further reading (Shebl et al., 2012; Antonic et al., 2013; Cummins and Tangney, 2013; Sun and Kato, 2016; Pasquereau-Kotula et al., 2018).

In several mouse models of carcinogenesis, the GF animals or those treated with antibiotics were protected or showed reduced cancer development compared with the CONV-R counterparts (Dove et al., 1997; Uronis and Jobin, 2009). The CONV-R IL-10 deficient mice, that develop intestinal inflammation due to a blunted immune regulation, are highly susceptible to chemically induced cancer but protected when raised GF (Uronis and Jobin, 2009). Similarly, in the presence of a microbiota, the APC^Min/+^ mice develop a greater number of adenomatous polyps as to when reared GF (Dove et al., 1997). Furthermore, TLR activation promotes carcinogenesis as APC^Min/+^ mice, that are also deficient in MYD88, a key component of the signaling pathway sensing microbial components, develop less and smaller tumors (Rakoff-Nahoum and Medzhitov, 2007). However, dysregulated sensing of the microbiota can also promote carcinogenesis, as the cancer cells express high levels of Toll-like receptors and their activation by microbial products contributes to the growth and spread of tumor (Schwabe and Jobin, 2013). Finally, apart from solely “presenting” or secreting structural antigens, the bacteria can also modulate inflammation and carcinogenesis via their metabolic functions (Louis et al., 2014). Bacteria are required for the production of secondary bile acids, which have carcinogenic effects (Breuer and Goebell, 1985). Diets rich in fiber promote the growth of short-chain fatty acid-producing bacteria, which protect against colitis-associated colorectal cancer (Donohoe et al., 2014) via activation of the receptors, GPR109a and GPR43 (Tang et al., 2011; Singh et al., 2014).

Recent work performed with the fruit fly showed very similar dependencies. A tight connection between the local immune system, the microbiome, and tumor development seems to also exist in this simple model (Lee, 2009; Bangi, 2013; Panayidou and Apidianakis, 2013). Bacterial infection with *P. aeruginosa* can induce intestinal dysplasia by activation of JNK signaling, which leads to Ras expression in intestinal stem cells (Apidianakis et al., 2009). This synergistic and tumor promoting effect of JNK and Ras activation was observed in several studies. It suppresses apoptosis and leads to loss of cell polarity due to increased expression of matrix metalloproteinases (Uhlirova and Bohmann, 2006; Wu et al., 2010; Brumby et al., 2011). Importantly, Christofi and Apidianakis (2013) showed that this synergism of the pro-oncogenic Ras and JNK activities depends on the microbiota (Bangi et al., 2012). The role of inappropriate persistent activation of innate immune responses on the progenitor cell hyperplasia was corroborated in flies with Notch-dependent intestinal tumors. Interestingly, this persistent immune activation restricted to progenitor cells was not sufficient to alter the composition of the fly’s microbiota (Petkau et al., 2017).

The tight connection between the microbiota, the immune system, and intestinal tumorigenesis impacts tumor growth in two ways in vertebrates: Indirect tumor progression is mediated by factors such as CCL5 and cytokines like IL-17 and IL-23. Besides this, a direct influence could, for example, be observed by deoxycholic acid from *Clostridium* sp. or Colbactin from *E. coli* (Gagliani et al., 2014). Although this interaction is less well studied in *Drosophila*, it is possible that similar mechanisms are operative, especially as deoxycholic acid can be produced by *Clostridium perfringens*, which is one of the commensal microbes known to promote growth and development in the fly (Liu et al., 2016). Another possible connection between the microbiota, JNK signaling, and cancer progression was identified for the CagA protein, which is known as a virulence factor in *H. pylori*-induced diseases. Increased CagA levels directly induced the expression of the antimicrobial peptide *diptericin* and of the *dual oxidase*, leading to increased ROS production in the intestinal mucosa. Thus, the enhanced CagA levels synergistically foster proliferation in cell autonomous and non-cell autonomous ways, caused by alterations of the microbial communities (Jones et al., 2017). It is still a matter of debate if the microbiota influences the extra-intestinal cancer in humans (Dapito et al., 2012; Xuan et al., 2014). Recent work indeed showed that the intestinal bacteria and their metabolic activities affect the antitumor immune function and thereby carcinogenesis in the liver (Loo et al., 2017; Yu and Schwabe, 2017; Ma et al., 2018). Using *Drosophila* as a model, this question was addressed especially regarding putative interactions between the microbiota and the gut-brain-axis. Slight differences between the microbial communities of flies with and without cancer supported the idea of such an interdependency (Jacqueline et al., 2017). The interplay of the microbiome on cancer development is one of the main questions for further studies (Schroeder and Backhed, 2016) and *Drosophila* or mice promise to be informative models for this inquiry.

In sum, specific microbiota entities might shape the microbiome or produce metabolites that increase the inflammatory tone and thereby promote transformation of epithelial cells promoting tumorigenesis (Sears and Pardoll, 2011).

## Success of Cancer Therapy Also Depends on the Microbiota

Treatment efficacy of a given cancer therapy greatly varies among patients. Finding out why some patients successfully respond to treatment with a drug while it fails in others is an immense challenge of personalized medicine but also holds great promise. In the past years a growing body of evidence was accumulated, which linked microorganisms to the efficacy of cancer therapies (Zitvogel et al., 2015; Alexander et al., 2017). Many cancer treatments, such as radiation or chemotherapy, affect the microbiota composition and thus promote dysbiosis (Von Bultzingslowen et al., 2003; Nam et al., 2013). The gut microbiome carries a large gene arsenal that encodes highly diverse metabolic functions (Sommer and Backhed, 2013). These bacterial enzymes not only assist in the digestion of dietary nutrients but also metabolize xenobiotics (Maurice et al., 2013; Koppel et al., 2017) and drugs (Koropatkin and Martens, 2017; Wu et al., 2017), which in some cases can render the substance inactive or in contrast can be required for its functionality in the host. For example, it is known that the bacterium *Mycoplasma hyorhinis* can metabolize the chemotherapeutic drug gemcitabine, a nucleoside analog (2′,2′-difluorodeoxycytidine) used to treat patients with various cancers, into its deaminated inactive form 2′,2′-difluorodeoxyuridine (Plunkett et al., 1995; Vande Voorde et al., 2014; Lehouritis et al., 2015). In a recent paper, Geller et al. (2017) showed that only certain bacteria expressing the enzyme cytidine deaminase (CDDL), which is mainly carried by γ-proteobacteria, are capable of modifying gemcitabine. More importantly, when mice were infected with these CDDL-positive bacteria and subjected to a colon cancer model, the tumors became resistant to gemcitabine and this chemoresistance could be rescued by antibiotic treatment. In humans, several cancer types are routinely treated with gemcitabine but also contain CDDL-positive bacteria, which suggests that the treatment efficacy might be increased by the addition of antibiotics (Geller et al., 2017). *F. nucleatum* is not only enriched in tumor tissue but also promotes cancer chemoresistance to treatment with oxaliplatin, a platinum compound inhibiting DNA synthesis and causing cell death (Graham et al., 2004), or 5-fluorouracil, a thymidylate synthase inhibitor blocking the synthesis of thymidine and DNA replication (Longley et al., 2003). Yu et al. (2017) recently elucidated the underlying molecular pathway. *Fusobacterium* downregulates the expression of the two miRNAs, miRNA-18a^∗^ and miRNA-4802, via a TLR4/MYD88-dependent-mechanism and thereby inhibits apoptosis of cancer cells in favor of autophagy.

The gut microbiota, however, does not only promote chemoresistance but can also improve treatment efficacy or even be essential for treatment success. The antitumor effects of oxaliplatin were markedly reduced in GF or antibiotic-treated mice. Mechanistically, the microbiota activates tumor-infiltrating myeloid cells at least in part via the TLR4-MYD88 signaling pathway leading to the production of cytotoxic reactive oxygen species (Iida et al., 2013). Similarly, the antitumor efficacy of cyclophosphamide therapy was dependent on the microbiota, in particular the Gram-positive bacteria, which include *Lactobacillus johnsonii*, *Lactobacillus murinus*, *Enterococcus hirae*, and segmented filamentous bacteria, as well as the Gram-negative bacterium *Barnesiella intestinihominis* (Viaud et al., 2013; Daillere et al., 2016). Cyclophosphamide alters the intestinal microbiota composition and induces translocation of *E. hirae* into secondary lymphoid organs, where it drives the differentiation of proinflammatory T helper cells (Th17 and Th1 cells) (Viaud et al., 2013). *Barnesiella intestinihominis*, however, accumulates in the colon and promotes the infiltration of IFN-gamma-producing γδ-T cells in cancer lesions (Daillere et al., 2016). This combination produces a proinflammatory tone, which confers the inflammatory antitumor response of cyclophosphamide. Tumor-bearing mice raised GF or treated with antibiotics showed a reduction in proinflammatory T cell responses and their tumors were resistant to cyclophosphamide (Viaud et al., 2013). Notably, the immune sensor Nucleotide-binding oligomerization domain-containing protein 2 (NOD2), which recognizes the components of the bacterial cell wall, limits bacterial translocation and accumulation and thereby cyclophosphamide’s anticancer activity (Daillere et al., 2016).

The cancer-modulating effects of the microbiota are not restricted to classical chemotherapy but also extend to novel cancer immunotherapy targeting specific molecules. Antibodies targeting cytotoxic T-lymphocyte-associated protein 4 (CTLA-4) function act as potent cancer immunotherapeutics, but the tumors did not respond to CTLA blockade in antibiotic-treated or GF mice (Vetizou et al., 2015). The antitumor effects of the CTLA-4 blockade depended on extracellular polysaccharides of the two *Bacteroides* species *B. thetaiotaomicron* and *B. fragilis* via activation of T cells. Thus, *B. fragilis* can promote carcinogenesis (Goodwin et al., 2011; Boleij et al., 2015) and at the same time also support cancer immunotherapy (Vetizou et al., 2015). These seemingly contradictory effects of *B. fragilis* on cancer could be due to its pleiotropic effects on the epithelial and immune cells, either promoting epithelial transformation or boosting protective immune responses. Further studies are, however, required to disentangle the functions of *B. fragilis* for cancer development and treatment. Antibodies against programmed cell death 1 ligand 1 (PD-L1) are another commonly used cancer immunotherapy that is dependent on the microbiota. Treatment efficacy and antitumor T-cell responses of PD-L1 blockade were increased in mice that harbored a microbiota rich in Bifidobacteria as these stimulated the generation of tumor-specific T cells that led to an increase of T cells in the tumor (Sivan et al., 2015). The anticancer effects of anti-PD-L1 antibodies were abolished in the GF mice, by heat inactivation of the bacteria or depletion of CD8-positive T cells. The efficacy of the anti-PD-L1 therapy also seems to be correlated with the commensal microbiota in human melanoma patients. An association was observed between the composition of the fecal microbiota of patients that responded to anti-PD-L1 therapy and patients who did not respond to this therapy. Fecal transplantation from responders into GF mice exhibited an increased anti-PD-L1 therapeutic response by showing decreased tumor growth and higher levels of CD8-positive T cells compared to those mice, which were reconstituted with non-responder fecal material (Matson et al., 2018). Another study showed that the responders among melanoma patients have a higher gut-associated bacterial diversity after initiation of an anti-PD1 therapy compared to non-responders. Interestingly, an enrichment of *Faecalibacterium* of the order Clostridiales in responders was detected whereas in non-responders members of the Bacteroidales were expanded. The enrichment of *Faecalibacterium* correlated with prolonged survival and higher levels of CD8-positive T cells in the tumor microenvironment. Moreover, the commensal microbiota of responders is associated with anabolic functions, which may influence the immune response of the host. Fecal transplantation from responding and non-responding patients in germ-free mice recapitulated these results (Gopalakrishnan et al., 2018). Mice transplanted with responder fecal microbiome showed an improved response to anti-PD-L1 therapy, reduced tumor size, and higher levels of CD8-positive T cells compared to the recipient mice with non-responder fecal microbiome (Gopalakrishnan et al., 2018).

Taken together, the microbiota metabolizes chemotherapeutics and thereby modulates their functionality and ultimately the efficacy of chemotherapy.

## Conclusion

**FIGURE 1:**
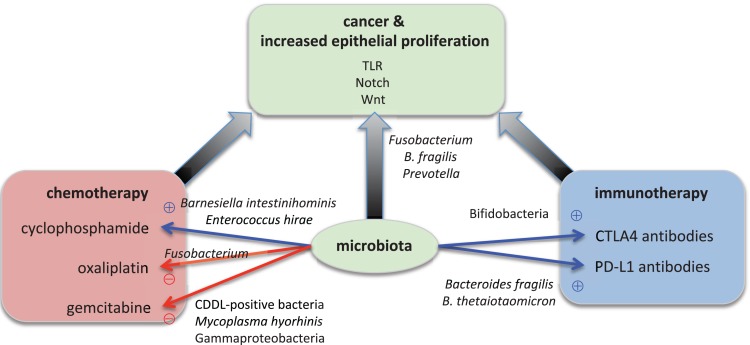
Microbial effects on epithelial proliferation, carcinogenesis, and cancer therapy. Specific microorganisms contribute to carcinogenesis. For example, *Fusobacterium nucleatum, Bacteroides fragilis*, and *Prevotella* species boost the epithelial proliferation. Bacterial signals are transmitted through the TLR (Toll-like receptor), Notch, and Wnt signaling pathways in the epithelium. The overactive proliferation then leads to cancer development. Specific microorganisms also modulate the success of cancer therapy. On the one hand, for example, *Barnesiella intestinihominis* and *Enterococcus hirae* are essential for the anti-tumor function of oxaliplatin whereas on the other hand Bifidobacteria, *Bacteroides fragilis* and *B. thetaiotaomicron* are required for the anti-tumor function of immunotherapy with antibodies against CTLA4 and PD-L1, respectively. On the other hand, *Mycoplasma hyorhinis* and CDDL-positive bacteria such as Gammaproteobacteria inactivate the chemotherapeutic agent gemcitabine, and *Fusobacterium* renders cyclophosphamide non-functional.

Microorganisms affect epithelial homeostasis, development of carcinoma, and the response to cancer therapy (**Figure 1** and **Supplementary Table S1**). We have begun to identify specific bacteria and molecular pathways that either confer protection from tumorigenesis or promote cancer development and confer resistance to chemotherapy. Yet, the current data also points toward the fact that single microorganisms are likely not sufficient to cause cancer on their own, but instead require additional cues from the host (e.g., immune system components), the environment (e.g., dietary mutagens), or other microorganisms (potentiation effects) for a functional carcinogenesis. In a diverse microbial world, this may not only be related to the differential abundance of specific taxa such as *F. nucleatum*, but also associated with metabolic principles, which can be mediated by a wider array of bacteria with similar metabolic activities. Epithelial cancer development could also be interpreted as an evolutionary process, where it will be of interest to determine the levels of selective advantages on both sides–the cancerous tissue as the novel host and the novel cancer-associated consortia. The findings are consistent with the view that an organism is protected by both its own immune system and the components of the microbiota (“co-immunity”) (Chiu et al., 2017) and that the immune system evolved to control the natural microbiota rather than to defend against pathogens (McFall-Ngai, 2007; Bosch, 2014). Model organisms, such as the mouse and *Drosophila*, represent highly informative experimental systems to transition from correlation to the elucidation of functional interactions between the host and its associated microorganisms (McFall-Ngai et al., 2013). For example, *Drosophila* is a potential high throughput *in vivo* model to study the impact of the microbiota on the efficacy of cancer chemotherapeutics (Markstein et al., 2014). The fundamental host physiological processes including cell cycle regulation, immune regulation, or signaling pathways were identified and molecularly defined in these model organisms due to their ease of experimental manipulation and reduced complexity compared to humans. Similarly, these systems promise to elucidate the fundamental principles of host–microbiota interactions, despite the fact that the microbiota composition differs between humans and these model organisms, which limits a 1:1 translation of effects of specific bacteria among systems. Nevertheless, these novel findings provide new avenues to prevent epithelial carcinogenesis, to facilitate earlier diagnosis, and to improve the efficacy of chemotherapy by selective manipulation of the gut microbiota and personalized therapeutic approaches (Zhang et al., 2011; Blumberg and Powrie, 2012).

## Author Contributions

FS conceived the ideas and concepts of the manuscript. All authors wrote, reviewed, and approved the manuscript.

## Conflict of Interest Statement

The authors declare that the research was conducted in the absence of any commercial or financial relationships that could be construed as a potential conflict of interest.

## Supplementary Material

TABLE S1 Summary of effects of specific microorganisms on cancer. This table provides an overview of the known functions of specific microorganisms for cancer development or treatment of the respective hosts–human, mouse, and *Drosophila*.
