# Supplementary material for: Grow With the Challenge – Microbial Effects on Epithelial Proliferation, Carcinogenesis, and Cancer Therapy
Source: Front Microbiol. 2018 Sep 20;9:2020. doi: 10.3389/fmicb.2018.02020 (PMC6159313; doi:10.3389/fmicb.2018.02020)
Supplement: TABLE S1 — Summary of effects of specific microorganisms on cancer. This table provides an overview of the known functions of specific microorganisms for cancer development or treatment of the respective hosts–human, mouse, and Drosophila. [file Table_1.DOCX]

|  | **Microorganism** | **Host** | **Role in cancer** | **Function** | **Reference** |
| --- | --- | --- | --- | --- | --- |
| **Gram negative** | ***Helicobacter pylori*** | Human | Promotes cancer | CagA impairs tight junctions components and disrupts epithelial cell polarity (*in vitro).* | Wu et al., 2013  Hong et al., 2012  Zhang et al., 2012  Amieva et al., 2003  Saadat et al., 2007 |
|  |  | Mouse | Promotes neoplasia | Transgenic expression of CagA deregulates SHP-2, which in turn leads to abnormal proliferation. | Fox et al., 2003 |
|  |  | *Drosophila* | Promotes intestinal hyperplasia | Transgenic expression of CagA induces excessive intestinal cell proliferation and alters host microbiota. | Jones et al., 2017 |
|  | ***Fusobacterium nucleatum*** | Human | Promotes cancer/chemoresistance | FadA adhesin binds to E-cadherin to stimulate Wnt/ß-catenin signaling. Inhibits apoptosis of cancer cells by inducing genomic loss of miR-18a* and miR-4802 via the TLR4/MYD88 pathway. | Rubinstein et al., 2013  Yu et al., 2017 |
|  |  | Mouse | Promotes cancer/chemoresistance | Activates the TLR4/MYD88 signaling pathway, which leads to miR21 expression accompanied by reduced levels of the Ras GTPase RASA1. Attracts myeloid-derived suppressor cells. | Yang et al., 2017  Kostic et al., 2013 |
|  | ***Bacteroides fragilis*** | Human | Promotes cancer/chemotherapy | Mucosa and stool of colorectal cancer patients are associated with increased amount of the *Bacteroides fragilis* toxin. Immunostimulatory effect on CTLA-4 blockade. | Boleij et al., 2011  Toprak et al., 2006  Vetizou et al., 2015 |
|  |  | Mouse | Promotes cancer/chemotherapy | Produces toxin, resulting in spermin oxidase-dependent ROS production, which in turn leads to DNA damage. Immunostimulatory effect on CTLA-4 blockade. | Goodwin et al., 2011  Vetizou et al., 2015 |
|  | ***Salmonella*** | Human | Promotes cancer | AvrA protein was found in colorectal tissue. AvrA blocks ß-catenin degradation and attenuates NF-κB activity in human epithelial cells (*in vitro*). | Lu et al., 2017  Gonzalez-Escobedo et al., 2013  Mughini-Gras et al., 2018  Ye et al., 2007 |
|  |  | Mouse | Promotes cancer | AvrA protein was found in colorectal tissue. AvrA blocks ß-catenin degradation and thereby stimulates *c-myc* expression. AvrA induces cell proliferation and inhibits apoptosis. | Lu et al., 2017  Ye et al., 2007 |
|  | ***Pseudomonas aeruginosa*** | *Drosophila* | Promotes intestinal hyperplasia | JNK pathway dependent activation of Ras in intestinal stem cells. | Apidianakis et al., 2009 |
|  | ***Pseudomonas entomophila*** | *Drosophila* | Promotes intestinal hyperplasia | Provokes perturbation of the intestinal epithelium via the pore-forming toxin Monalysin and induces JNK pathway activity. | Vodovar et al., 2005  Opota et al., 2011 |
|  | ***Erwinia carotovara*** | *Drosophila* | Promotes intestinal dysplasia | Duox-dependent ROS-production. Excessive cell proliferation induced via JNK and JAK/STAT signaling. | Buchon et al., 2009 |
|  | ***Escherichia coli NC101*** | Mouse | Promotes cancer | The bacterial derived genotoxin Colibactin can cause DNA damage. | Arthur et al., 2012 |
|  | ***Prevotellaceae*** | Mouse | Promotes cancer | Promotes induction of CCL5-driven inflammation, which promotes epithelial cell proliferation via IL-6 signaling. | Elinav et al., 2011  Hu et al., 2013 |
|  | ***Klebsiella pneumoniae*** | Human | Promotes cancer | Pyogenic liver abscess with *Klebsiella pneumoniae* is associated with colorectal cancer | Huang et al., 2012 |
| **Gram positive** | ***Clostridium sp.*** | Mouse | Promotes cancer | The bacterial metabolite deoxycholic acid can cause DNA damage. | Yoshimoto et al., 2013 |
|  | ***Enterococcus faecalis*** | Human | Promotes cancer/beneficial for cancer treatment | Induces H_2_O_2_ production, which can cause DNA damage and activates EGFR signaling via H_2_O_2_ production (*in vitro*). Downregulates FIAF gene, which is associated with cancer development (*in vitro*). | Winters et al., 1998  Balamurugan et al., 2008  Boonanantanasarn et al., 2012  Grootaert et al., 2011 |
|  |  | Mouse | Promotes cancer/beneficial for cancer treatment | Activates Wnt/ß-catenin signaling in colon cells. Heat-killed *E. faecalis* EC12 suppresses Wnt/ß-catenin signaling and thereby reduces tumor development. | Wang et al., 2017  Miyamoto et al., 2017 |
|  | ***Streptococcus bovis/gallolyticus*** | Human | Promotes cancer | Induces expression of IL-1, IL-8 and COX-2. | Corredoira-Sanchez et al., 2012  Abdulamir et al., 2009  Abdulamir et al., 2010 |
|  |  | Mouse | Promotes cancer | Induces COX-2 expression. | Abdulamir et al., 2010 |
|  | ***Mycobacterium hyorhinis*** | Mouse | Promotes chemoresistance | Metabolizes gemcitabine and thereby inactivates the cytostatic activity | Vande Voorde et al., 2014 |
|  | ***Lactobacillus johnsonii*** | Mouse | Promotes chemotherapy | Translocate into secondary lymph organs in response to cyclophosphamide, where it drives the differentiation of naive CD4 positive T cells towards Th1 or Th17 cells. | Viaud et al., 2013 |
|  | ***Barnesiella intestinihominis*** | Mouse | Promotes chemotherapy | Accumulates in the colon in response to cyclophosphamide and increased the recruitment of IFN-$\gamma$ -producing γδ-T. | Daillere et al., 2016 |
|  | ***Faecalibacterium*** | Human | Promotes chemotherapy | Improves the anti-PD-L1 chemotherapy and the CD8-positive T cell function. | Matson et al., 2018  Gopalakrishnan et al., 2018 |
|  |  | Mouse |  |  |  |

**References:**

Abdulamir, A.S., Hafidh, R.R., and Bakar, F.A. (2010). Molecular detection, quantification, and isolation of Streptococcus gallolyticus bacteria colonizing colorectal tumors: inflammation-driven potential of carcinogenesis via IL-1, COX-2, and IL-8. *Mol Cancer* 9**,** 249. doi: 10.1186/1476-4598-9-249.

Abdulamir, A.S., Hafidh, R.R., Mahdi, L.K., Al-jeboori, T., and Abubaker, F. (2009). Investigation into the controversial association of Streptococcus gallolyticus with colorectal cancer and adenoma. *BMC Cancer* 9**,** 403. doi: 10.1186/1471-2407-9-403.

Amieva, M.R., Vogelmann, R., Covacci, A., Tompkins, L.S., Nelson, W.J., and Falkow, S. (2003). Disruption of the epithelial apical-junctional complex by Helicobacter pylori CagA. *Science* 300(5624)**,** 1430-1434. doi: 10.1126/science.1081919.

Apidianakis, Y., Pitsouli, C., Perrimon, N., and Rahme, L. (2009). Synergy between bacterial infection and genetic predisposition in intestinal dysplasia. *Proc Natl Acad Sci U S A* 106(49)**,** 20883-20888. doi: 10.1073/pnas.0911797106.

Arthur, J.C., Perez-Chanona, E., Muhlbauer, M., Tomkovich, S., Uronis, J.M., Fan, T.J., et al. (2012). Intestinal inflammation targets cancer-inducing activity of the microbiota. *Science* 338(6103)**,** 120-123. doi: 10.1126/science.1224820.

Balamurugan, R., Rajendiran, E., George, S., Samuel, G.V., and Ramakrishna, B.S. (2008). Real-time polymerase chain reaction quantification of specific butyrate-producing bacteria, Desulfovibrio and Enterococcus faecalis in the feces of patients with colorectal cancer. *J Gastroenterol Hepatol* 23(8 Pt 1)**,** 1298-1303. doi: 10.1111/j.1440-1746.2008.05490.x.

Boleij, A., van Gelder, M.M., Swinkels, D.W., and Tjalsma, H. (2011). Clinical Importance of Streptococcus gallolyticus infection among colorectal cancer patients: systematic review and meta-analysis. *Clin Infect Dis* 53(9)**,** 870-878. doi: 10.1093/cid/cir609.

Boonanantanasarn, K., Gill, A.L., Yap, Y., Jayaprakash, V., Sullivan, M.A., and Gill, S.R. (2012). Enterococcus faecalis enhances cell proliferation through hydrogen peroxide-mediated epidermal growth factor receptor activation. *Infect Immun* 80(10)**,** 3545-3558. doi: 10.1128/IAI.00479-12.

Buchon, N., Broderick, N.A., Chakrabarti, S., and Lemaitre, B. (2009). Invasive and indigenous microbiota impact intestinal stem cell activity through multiple pathways in *Drosophila*. *Genes Dev* 23(19)**,** 2333-2344. doi: 23/19/2333 [pii]

10.1101/gad.1827009.

Corredoira-Sanchez, J., Garcia-Garrote, F., Rabunal, R., Lopez-Roses, L., Garcia-Pais, M.J., Castro, E., et al. (2012). Association between bacteremia due to Streptococcus gallolyticus subsp. gallolyticus (Streptococcus bovis I) and colorectal neoplasia: a case-control study. *Clin Infect Dis* 55(4)**,** 491-496. doi: 10.1093/cid/cis434.

Daillere, R., Vetizou, M., Waldschmitt, N., Yamazaki, T., Isnard, C., Poirier-Colame, V., et al. (2016). Enterococcus hirae and Barnesiella intestinihominis Facilitate Cyclophosphamide-Induced Therapeutic Immunomodulatory Effects. *Immunity* 45(4)**,** 931-943. doi: 10.1016/j.immuni.2016.09.009.

Elinav, E., Strowig, T., Kau, A.L., Henao-Mejia, J., Thaiss, C.A., Booth, C.J., et al. (2011). NLRP6 inflammasome regulates colonic microbial ecology and risk for colitis. *Cell* 145(5)**,** 745-757. doi: 10.1016/j.cell.2011.04.022.

Fox, J.G., Rogers, A.B., Ihrig, M., Taylor, N.S., Whary, M.T., Dockray, G., et al. (2003). Helicobacter pylori-associated gastric cancer in INS-GAS mice is gender specific. *Cancer Res* 63(5)**,** 942-950.

Gonzalez-Escobedo, G., La Perle, K.M., and Gunn, J.S. (2013). Histopathological analysis of Salmonella chronic carriage in the mouse hepatopancreatobiliary system. *PLoS One* 8(12)**,** e84058. doi: 10.1371/journal.pone.0084058.

Goodwin, A.C., Destefano Shields, C.E., Wu, S., Huso, D.L., Wu, X., Murray-Stewart, T.R., et al. (2011). Polyamine catabolism contributes to enterotoxigenic Bacteroides fragilis-induced colon tumorigenesis. *Proc Natl Acad Sci U S A* 108(37)**,** 15354-15359. doi: 10.1073/pnas.1010203108.

Gopalakrishnan, V., Spencer, C.N., Nezi, L., Reuben, A., Andrews, M.C., Karpinets, T.V., et al. (2018). Gut microbiome modulates response to anti-PD-1 immunotherapy in melanoma patients. *Science* 359(6371)**,** 97-103. doi: 10.1126/science.aan4236.

Grootaert, C., Van de Wiele, T., Van Roosbroeck, I., Possemiers, S., Vercoutter-Edouart, A.S., Verstraete, W., et al. (2011). Bacterial monocultures, propionate, butyrate and H2O2 modulate the expression, secretion and structure of the fasting-induced adipose factor in gut epithelial cell lines. *Environ Microbiol* 13(7)**,** 1778-1789. doi: 10.1111/j.1462-2920.2011.02482.x.

Hong, S.N., Lee, S.M., Kim, J.H., Lee, T.Y., Kim, J.H., Choe, W.H., et al. (2012). Helicobacter pylori infection increases the risk of colorectal adenomas: cross-sectional study and meta-analysis. *Dig Dis Sci* 57(8)**,** 2184-2194. doi: 10.1007/s10620-012-2245-x.

Hu, B., Elinav, E., Huber, S., Strowig, T., Hao, L., Hafemann, A., et al. (2013). Microbiota-induced activation of epithelial IL-6 signaling links inflammasome-driven inflammation with transmissible cancer. *Proc Natl Acad Sci U S A* 110(24)**,** 9862-9867. doi: 10.1073/pnas.1307575110.

Huang, W.K., Chang, J.W., See, L.C., Tu, H.T., Chen, J.S., Liaw, C.C., et al. (2012). Higher rate of colorectal cancer among patients with pyogenic liver abscess with Klebsiella pneumoniae than those without: an 11-year follow-up study. *Colorectal Dis* 14(12)**,** e794-801. doi: 10.1111/j.1463-1318.2012.03174.x.

Jones, T.A., Hernandez, D.Z., Wong, Z.C., Wandler, A.M., and Guillemin, K. (2017). The bacterial virulence factor CagA induces microbial dysbiosis that contributes to excessive epithelial cell proliferation in the *Drosophila* gut. *PLoS Pathog* 13(10)**,** e1006631. doi: 10.1371/journal.ppat.1006631.

Kostic, A.D., Chun, E., Robertson, L., Glickman, J.N., Gallini, C.A., Michaud, M., et al. (2013). Fusobacterium nucleatum potentiates intestinal tumorigenesis and modulates the tumor-immune microenvironment. *Cell Host Microbe* 14(2)**,** 207-215. doi: 10.1016/j.chom.2013.07.007.

Lu, R., Bosland, M., Xia, Y., Zhang, Y.G., Kato, I., and Sun, J. (2017). Presence of Salmonella AvrA in colorectal tumor and its precursor lesions in mouse intestine and human specimens. *Oncotarget* 8(33)**,** 55104-55115. doi: 10.18632/oncotarget.19052.

Matson, V., Fessler, J., Bao, R., Chongsuwat, T., Zha, Y., Alegre, M.L., et al. (2018). The commensal microbiome is associated with anti-PD-1 efficacy in metastatic melanoma patients. *Science* 359(6371)**,** 104-108. doi: 10.1126/science.aao3290.

Miyamoto, S., Komiya, M., Fujii, G., Hamoya, T., Nakanishi, R., Fujimoto, K., et al. (2017). Preventive Effects of Heat-Killed Enterococcus faecalis Strain EC-12 on Mouse Intestinal Tumor Development. *Int J Mol Sci* 18(4). doi: 10.3390/ijms18040826.

Mughini-Gras, L., Schaapveld, M., Kramers, J., Mooij, S., Neefjes-Borst, E.A., Pelt, W.V., et al. (2018). Increased colon cancer risk after severe Salmonella infection. *PLoS One* 13(1)**,** e0189721. doi: 10.1371/journal.pone.0189721.

Opota, O., Vallet-Gely, I., Vincentelli, R., Kellenberger, C., Iacovache, I., Gonzalez, M.R., et al. (2011). Monalysin, a novel ss-pore-forming toxin from the *Drosophila* pathogen Pseudomonas entomophila, contributes to host intestinal damage and lethality. *PLoS Pathog* 7(9)**,** e1002259. doi: 10.1371/journal.ppat.1002259.

Rubinstein, M.R., Wang, X., Liu, W., Hao, Y., Cai, G., and Han, Y.W. (2013). Fusobacterium nucleatum promotes colorectal carcinogenesis by modulating E-cadherin/beta-catenin signaling via its FadA adhesin. *Cell Host Microbe* 14(2)**,** 195-206. doi: 10.1016/j.chom.2013.07.012.

Saadat, I., Higashi, H., Obuse, C., Umeda, M., Murata-Kamiya, N., Saito, Y., et al. (2007). Helicobacter pylori CagA targets PAR1/MARK kinase to disrupt epithelial cell polarity. *Nature* 447(7142)**,** 330-333. doi: 10.1038/nature05765.

Toprak, N.U., Yagci, A., Gulluoglu, B.M., Akin, M.L., Demirkalem, P., Celenk, T., et al. (2006). A possible role of Bacteroides fragilis enterotoxin in the aetiology of colorectal cancer. *Clin Microbiol Infect* 12(8)**,** 782-786. doi: CLM1494 [pii]

10.1111/j.1469-0691.2006.01494.x.

Vande Voorde, J., Sabuncuoglu, S., Noppen, S., Hofer, A., Ranjbarian, F., Fieuws, S., et al. (2014). Nucleoside-catabolizing enzymes in mycoplasma-infected tumor cell cultures compromise the cytostatic activity of the anticancer drug gemcitabine. *J Biol Chem* 289(19)**,** 13054-13065. doi: 10.1074/jbc.M114.558924.

Vetizou, M., Pitt, J.M., Daillere, R., Lepage, P., Waldschmitt, N., Flament, C., et al. (2015). Anticancer immunotherapy by CTLA-4 blockade relies on the gut microbiota. *Science* 350(6264)**,** 1079-1084. doi: 10.1126/science.aad1329.

Viaud, S., Saccheri, F., Mignot, G., Yamazaki, T., Daillere, R., Hannani, D., et al. (2013). The intestinal microbiota modulates the anticancer immune effects of cyclophosphamide. *Science* 342(6161)**,** 971-976. doi: 10.1126/science.1240537.

Vodovar, N., Vinals, M., Liehl, P., Basset, A., Degrouard, J., Spellman, P., et al. (2005). *Drosophila* host defense after oral infection by an entomopathogenic Pseudomonas species. *Proc Natl Acad Sci U S A* 102(32)**,** 11414-11419. doi: 10.1073/pnas.0502240102.

Wang, X., Yang, Y., and Huycke, M.M. (2017). Commensal-infected macrophages induce dedifferentiation and reprogramming of epithelial cells during colorectal carcinogenesis. *Oncotarget* 8(60)**,** 102176-102190. doi: 10.18632/oncotarget.22250.

Winters, M.D., Schlinke, T.L., Joyce, W.A., Glore, S.R., and Huycke, M.M. (1998). Prospective case-cohort study of intestinal colonization with enterococci that produce extracellular superoxide and the risk for colorectal adenomas or cancer. *Am J Gastroenterol* 93(12)**,** 2491-2500. doi: 10.1111/j.1572-0241.1998.00710.x.

Wu, Q., Yang, Z.P., Xu, P., Gao, L.C., and Fan, D.M. (2013). Association between Helicobacter pylori infection and the risk of colorectal neoplasia: a systematic review and meta-analysis. *Colorectal Dis* 15(7)**,** e352-364. doi: 10.1111/codi.12284.

Yang, Y., Weng, W., Peng, J., Hong, L., Yang, L., Toiyama, Y., et al. (2017). Fusobacterium nucleatum Increases Proliferation of Colorectal Cancer Cells and Tumor Development in Mice by Activating Toll-Like Receptor 4 Signaling to Nuclear Factor-kappaB, and Up-regulating Expression of MicroRNA-21. *Gastroenterology* 152(4)**,** 851-866 e824. doi: 10.1053/j.gastro.2016.11.018.

Ye, Z., Petrof, E.O., Boone, D., Claud, E.C., and Sun, J. (2007). Salmonella effector AvrA regulation of colonic epithelial cell inflammation by deubiquitination. *Am J Pathol* 171(3)**,** 882-892. doi: 10.2353/ajpath.2007.070220.

Yoshimoto, S., Loo, T.M., Atarashi, K., Kanda, H., Sato, S., Oyadomari, S., et al. (2013). Obesity-induced gut microbial metabolite promotes liver cancer through senescence secretome. *Nature* 499(7456)**,** 97-101. doi: 10.1038/nature12347.

Yu, T., Guo, F., Yu, Y., Sun, T., Ma, D., Han, J., et al. (2017). Fusobacterium nucleatum Promotes Chemoresistance to Colorectal Cancer by Modulating Autophagy. *Cell* 170(3)**,** 548-563 e516. doi: 10.1016/j.cell.2017.07.008.

Zhang, Y., Hoffmeister, M., Weck, M.N., Chang-Claude, J., and Brenner, H. (2012). Helicobacter pylori infection and colorectal cancer risk: evidence from a large population-based case-control study in Germany. *Am J Epidemiol* 175(5)**,** 441-450. doi: 10.1093/aje/kwr331.
